# Familial dysalbuminemic hyperthyroxinemia combined with Graves’ disease: a rare case report

**DOI:** 10.1186/s12902-023-01481-5

**Published:** 2023-10-18

**Authors:** Yuanmeng Li, Yue Chi, Xiaofeng Chai, He Liu, Naishi Li, Xiaolan Lian

**Affiliations:** 1 Department of Endocrinology, Key Laboratory of Endocrinology of National Health Commission, Peking Union Medical College Hospital, Chinese Academy of Medical Sciences & Peking Union Medical College, Beijing, China; 2 State Key Laboratory of Complex Severe and Rare Diseases, Peking Union Medical College Hospital, Chinese Academy of Medical Sciences & Peking Union Medical College, Beijing, China; 3 Department of Medical Records, Peking Union Medical College Hospital, Chinese Academy of Medical Sciences & Peking Union Medical College, Beijing, China; 4 WHO Family of International Classifications Collaborating Center of China, Beijing, China

**Keywords:** Familial dysalbuminemic hyperthyroxinemia, Graves’ disease, Syndromes of inappropriate secretion of thyroid stimulating hormone, Hypothyroidism, Case report

## Abstract

**Background:**

Familial dysalbuminemic hyperthyroxinemia (FDH) is an autosomal dominant disease characterised by an abnormally increased affinity of albumin for serum thyroxine. Assay interference and differential diagnosis remain challenging for FDH. The condition is more complicated when FDH is combined with primary thyroid diseases. Co-occurrence of FDH and Graves’ disease is rare.

**Case presentation:**

We report the case of a 28-year-old woman with complex FDH and coexisting Graves’ disease. Initially, the existence of FDH was not recognised. Graves’ disease was relieved after treatment with antithyroid drugs and two administrations of radioactive iodine therapy. She subsequently developed primary hypothyroidism and was prescribed levothyroxine replacement. However, thyroid function failed to normalise despite frequent levothyroxine dose adjustments. Ultimately, syndromes involving the inappropriate secretion of thyroid-stimulating hormone (IST) were considered, and FDH was successfully differentiated from other causes of IST.

**Conclusions:**

A greater focus on FDH when investigating the causes of IST is critical to correctly evaluate thyroid function status and avoid inappropriate treatment, especially in complicated cases with concurrent FDH and primary thyroid diseases.

## Background

Familial dysalbuminemic hyperthyroxinemia (FDH) is an autosomal dominant disorder caused by mutations in the albumin gene (*ALB)* that results in an abnormally increased affinity of albumin for serum thyroxine (T4). Patients with FDH are physiologically euthyroid despite of the increased levels of circulating total thyroxine (TT4). With an estimated prevalence of 1 in 10 000 individuals, FDH is the predominant form of euthyroid hyperthyroxinemia within the Caucasian population [1]. However, FDH is clinically underdiagnosed, in part because it can mimic the thyroid function profiles of thyroid hormone resistance syndrome (RTH) and thyroid stimulating hormone (TSH)-secreting pituitary adenomas (TSHomas), making the differential diagnosis difficult. A few cases of concurrent FDH and Graves’ disease (GD) have been reported. The coexistence of these two conditions further complicates the diagnosis and hinders appropriate treatment.

Herein, we describe a complex case of FDH combined with GD. After receiving antithyroid drugs and two rounds of radioactive iodine (RAI) therapy, GD was relived, and subsequent treatment for hypothyroidism was initiated. However, proper diagnosis and treatment were substantially delayed because of the complicated fluctuations in thyroid function.

The purpose of this article is to analyse the differentiation of FDH from RTH and TSHomas and to summarise the characteristics of FDH combined with GD.

## Case presentation

A 28-year-old Chinese woman was found to have abnormal thyroid function tests following routine testing in May 2017. The thyroid function test (Chemiluminescence immunoassay, Roche, one-step method) results were: free thyroxine (FT4) 1.97 ng/dL (reference range 0.58–1.64), free triiodothyronine (FT3) 4.42 pg/mL (reference range 2.39–4.2), TSH 2.060 μIU/mL (reference range 0.34–6.05). The patient did not receive any treatment since she had no thyrotoxic symptoms. Thyroid function tests (Chemiluminescence immunoassay, Siemens, one-step method) were repeated in June 2018 in a local laboratory: TT4 13.6 μg/dL (reference range 4.3–12.5), total triiodothyronine (TT3) 1.28 ng/mL (reference range 0.6–1.81), TSH 0.19 μIU/mL (reference range 0.35–5.5). Still, the patient did not visit any other hospitals because she had no symptoms. However, in March 2019, the patient developed palpitations, increased hunger, polyphagia, increased stool frequency, and weight loss. Thyroid function tests were repeated and listed as follows: FT4 3.48 ng/dL (reference range 0.7–1.48), FT3 16.65 pg/mL (reference range 1.58–3.91), TSH < 0.005 μIU/mL (reference range 0.35–4.94), and the anti-TSH receptor antibody (TRAb) level was significantly elevated (11.66 IU/L). GD was diagnosed, and the patient received methimazole therapy at a dose of 30 mg per day. The thyroid function tests were repeated when the thyrotoxicosis did not improve after one month: FT4 6.73 ng/dL (reference range 0.93–1.7), FT3 12.19 pg/mL (reference range 2.0–4.4), TSH < 0.005 μIU/mL (reference range 0.27–4.2). As the patient was concerned that a longer course of methimazole therapy might delay her pregnancy planning, she decided to change treatment. RAI therapy was initiated in May 2019. The RAI dose was 5 millicurie (mCi), mainly based on the weight of the thyroid estimated by nuclide scanning and the iodine uptake rate. Five months later, re-evaluation of thyroid function revealed a persistently elevated serum FT4 level and an enduring reduction in serum TSH level: FT4 2.13 ng/dL (reference range 0.93–1.7), FT3 4.4 pg/mL (reference range 2–4.4), TSH < 0.005μIU/mL. As the patient was anxious to prepare for pregnancy, she elected to discontinue observation and undergo a second round of RAI therapy, which was initiated in November 2019 with a dose of 4.62 mCi. Prednisone was not used during the two rounds of RAI therapy.

**Fig. 1:**
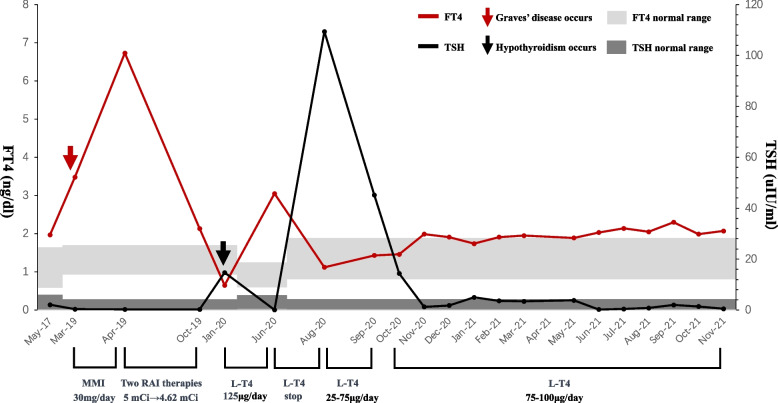
A summary of the patient’s clinical course. FT4, free thyroxine; TSH, thyroid stimulating hormone; MMI, methimazole; RAI, radioactive iodine; mCi, millicurie; L-T4, levothyroxine

In January 2020, the patient experienced fatigue and weight gain. Repeated thyroid function test revealed as follows: FT4 0.65 ng/dL (reference range 0.93–1.7), FT3 1.57 pg/mL (reference range 2–4.4), TSH 14.71 μIU/mL. At this point, she was prescribed levothyroxine with a dosage gradually increased to 125 μg (1.92 μg/kg) per day. The thyroid function test results in June 2020 were as follows: FT4 3.05 ng/dL (reference range 0.59–1.25), FT3 2.83 pg/mL (reference range 2.14–4.21), TSH 0.07 mIU/L (reference range 0.56–5.91). On advice at the local hospital, the patient stopped taking levothyroxine. One month later, the patient developed fatigue again and visited the outpatient department of our hospital. Levothyroxine replacement was restarted; however, the dosage required frequent adjustments because of difficulty achieving normal thyroid function test results. A summary of the patient’s clinical course is presented in Fig. 1.

**Fig. 2:**
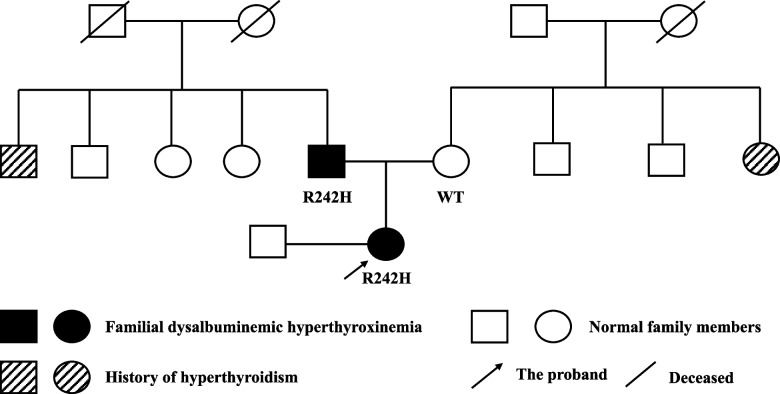
The family pedigree of the patient

The patient was healthy, married, and childless. The patient’s father had consistently abnormal thyroid tests in May 2020 and December 2021, with results as follows: In May 2020, FT4 was 36.24 pmol/L (reference range 12–22), FT3 was 6.4 pmol/L (reference range 3.1–6.8), and TSH was 1.98 μIU/mL (reference range 0.27–4.2). In December 2021, FT4 was 35.75 pmol/L (reference range 6.44–18.02), FT3 was 5.70 pmol/L (reference range 2.76–6.45), and TSH was 2.608 μIU/mL (reference range 0.35–5.1). The father remained asymptomatic and untreated. The patient’s mother had normal thyroid function. Additionally, an uncle and an aunt had a history of hyperthyroidism but achieved remission with methimazole treatment; their thyroid function test results in December 2021 were within normal limits (Fig. 2).

## Investigation

In December 2021, the patient was admitted to our hospital. Thyroid function tests were performed simultaneously using two different immunoassay methods (Centaur, one-step method; Abbott, two-step method), and the results were largely consistent (Table 1). The TRAb level was elevated to 10.48 IU/L (reference range < 2.5), whereas the antithyroid peroxidase antibody, antithyroglobulin antibody, and thyroglobulin levels were all within the normal ranges.

**Table 1:** Results of simultaneous thyroid function tests using different methods

	FT4	FT3	TT4	TT3	TSH	Detection date and treatment
One-step method (Centaur)	2.03 ng/dL (0.81–1.89)	4.55 pg/mL (1.8–4.1)	20.20 μg/dL (4.3–12.5)	1.34 ng/mL (0.66–1.92)	0.232 μIU/mL	Detected on 06/16/21, while on 1.43 μg/kg levothyroxine daily, 19 months following the second dose of the radioactive iodine
Two-step method (Abbott)	1.52 ng/dL (0.70–1.48)	1.06 ng/mL (0.58–1.59)	14.51 μg/dL (4.84–11.72)	3.46 pg/mL (1.71–3.71)	0.305 μIU/mL	

TSHomas and RTH were further investigated for the differential diagnosis. The exact data were listed as follows: sex hormone binding globulin (SHBG) 49.7 nmol/L (18.2–135.5), serum iron 61 μg/dL (50–170), ferritin 6 ng/mL (14–307). The levels of anterior pituitary hormones were within normal limits, and the dynamic contrast-enhanced magnetic resonance imaging (MRI) of the pituitary showed no signs of a pituitary tumour. In the somatostatin analogue test using Sandostatin (Novartis, Switzerland), the suppression ratios of TSH at 24 h versus 0 h and 2 h were determined to be 80.4% and 56.7%, respectively (Table 2).

**Table 2:** Results of the somatostatin analogue test

The somatostatin analogue test	0 h	2 h	4 h	6 h	8 h	24 h	48 h	72 h
TSH (μIU/mL)	1.041	0.471	0.307	0.252	0.329	0.204	0.235	0.244
FT3(pg/mL)	3.53	3.76	3.70	3.57	3.51	3.34	3.14	2.86
FT4(ng/dL)	1.91	2.10	1.91	1.74	1.92	1.68	1.62	1.68

Methods of the somatostatin analogue test: Somatostatin analogue was administered subcutaneously nine times at a dosage of 100 μg every 8 h. Serum TSH, FT3 and FT4 levels were measured at 0, 2, 4, 6, 8, 24, 48, and 72 h after the first injection

**Fig. 3:**
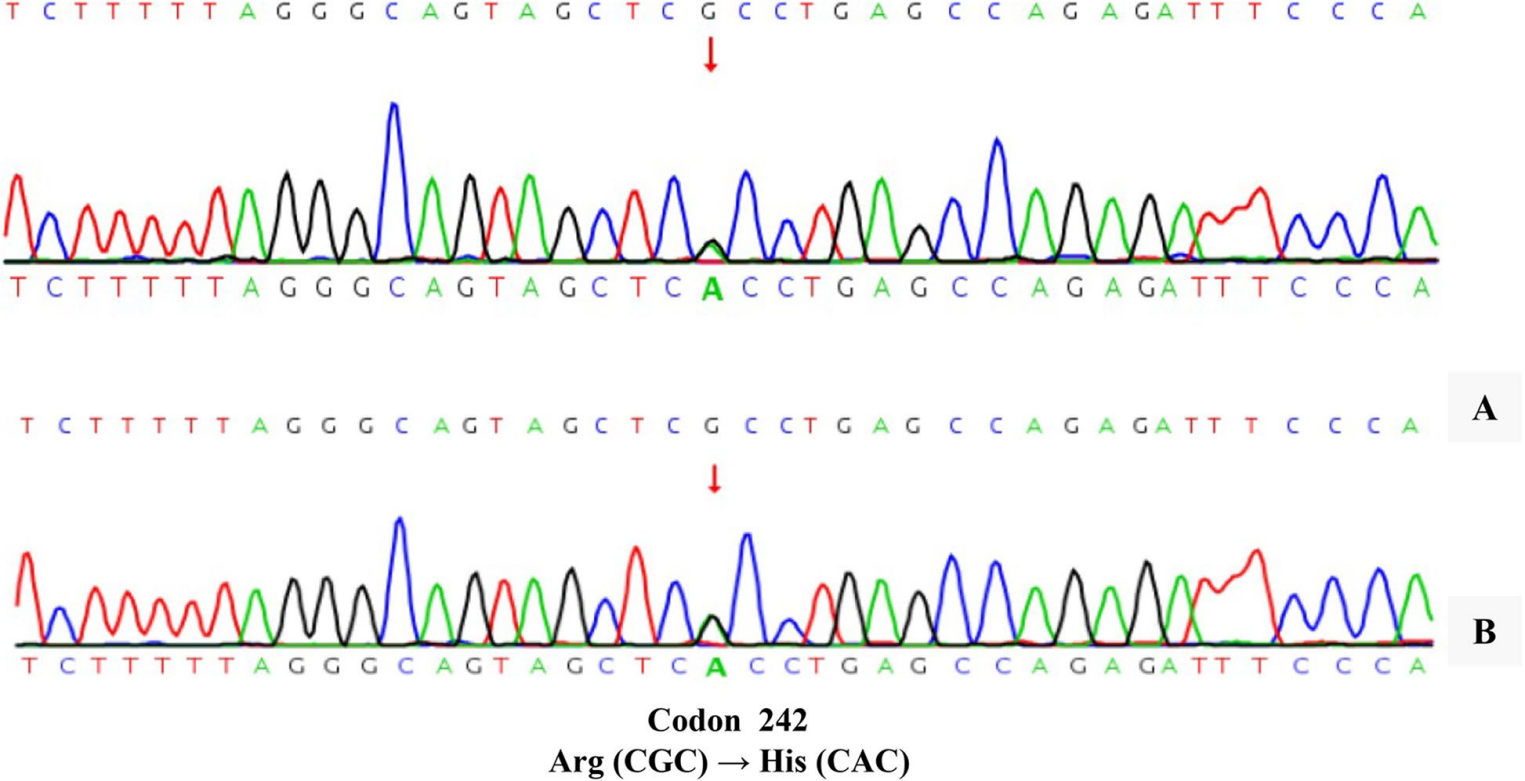
Gene sequencing of the patient (**A**). Confirmed results in the patient’s father by Sanger sequencing (**B**)

As the patient had no symptoms of hypermetabolism, showed normal levels of SHBG, and lacked signs of adenoma on pituitary MRI, the diagnosis of TSHomas was excluded, despite the supportive result of the somatostatin analogue test. Considering that the patient and her father had similar thyroid dysfunction, whole-exome sequencing was performed on the patient’s peripheral blood. A heterozygous mutation at exon 7 (c.725G > A) of the *ALB* gene was discovered (Fig. 3A), which was also verified in her father by Sanger sequencing (Fig. 3B).

## Discussion and conclusions

FDH was first reported by Hennemen et al. and Lee et al. in 1979 [2, 3]. Since then, the underlying pathogenesis of FDH has gradually been elucidated. Because of the increased affinity of mutant albumin for thyroxine, TT4 levels are elevated in patients with FDH. Theoretically, FT4 levels should not be affected. In fact, the FT4 value is characteristically within normal limits if direct assays such as equilibrium dialysis and ultrafiltration are used [4].

However, direct assays are rarely used in clinical practice because of limitations including high technical requirements and costs. Chemiluminescence immunoassay, a method that relies on the competition between T4 analogues and unbound T4 to determine the level of FT4, is the most commonly used technique. In patients with FDH, the chemiluminescence immunoassay is affected by the enhanced binding of mutant albumin to T4 analogues, resulting in falsely elevated FT4 values. Some studies have attempted to solve this problem by adding an additional washing step to avoid contact between T4 analogues and serum albumin, the so-called two-step method [5]. However, Khoo et al. revealed the potential for misleading results with falsely elevated FT4 levels using both one-step and two-step immunoassay methods [6]. Furthermore, they concluded that the varying susceptibility of FT4 to interference in different assays was likely attributable to differences in assay conditions and buffer composition, rather than being associated with assay architecture [6]. In the present case, circulating FT4 levels measured by the Centaur (one-step method) and Abbott (two-step method) methods were both elevated to a similar extent. These findings suggest that it is unreliable to assess the diagnosis of FDH solely by comparing changes in FT4 values measured using the one-step and two-step methods.

Diagnostic difficulties for FDH lie not only in the interference of detection assays but also in the differential diagnosis from other causes of the inappropriate secretion of thyroid-stimulating hormone (IST), especially RTH and TSHomas. RTH is an inherited disorder characterised by impaired sensitivity of targeted tissues to thyroid hormones. The majority of cases involve resistance to thyroid hormone β (RTHβ), caused by mutations in the thyroid hormone receptor β gene. FDH can mimic the typical thyroid function profile of RTH. By summarising the characteristics of RTHβ and FDH, Dieu et al. proposed that if a patient is asymptomatic and the circulating FT3 value is less than 1.11 times the upper limit of the reference range, the diagnosis is more likely FDH than RTHβ [7]. Their conclusions are applicable to the present case.

For patients suspected of having TSHomas, signs of adenoma on pituitary imaging and a positive somatostatin analogue test result can support the diagnosis. Han et al. proposed a cut-off point of 44.46% (suppression ratio of TSH at 24 h versus 2 h) for the differential diagnosis of TSHomas and other causes of IST (mainly RTHβ) [8]. In the case of our patient, the somatostatin analogue test was conducted with the intention of distinguishing between TSHomas and RTHβ. While the results initially reached the TSHomas diagnosis threshold, the ultimate diagnosis was FDH. Considering the expected normal TSH secretion in FDH patients, it is theoretically reasonable to expect that the somatostatin test results would resemble those of healthy individuals, even though there is no definitive data on how healthy individuals react to this test. Therefore, while the somatostatin test may be a valuable tool in distinguishing between TSHomas and RTHβ, its utility in identifying FDH may be limited.

In terms of incidence, RTH occurs in approximately 1 in 40 000 live births [9], and TSHomas are estimated to affect 1 in 1 000 000 individuals [10]. In contrast, FDH exhibits a relatively high incidence rate of 1 in 10 000 [1]. However, prior research efforts related to IST has predominantly focused on the identification of RTHβ and TSHomas. In fact, according to established guidelines, FDH should be excluded as a possibility before considering RTH and TSHomas [11]. Specifically, when faced with elevated thyroid hormone levels and non-suppressed TSH levels, the initial investigative step should involve the exclusion of potential methodological interferences. These interferences may include the presence of circulating heterophilic antibodies, anti-iodothyronine auto-antibodies, and anomalies of albumin (i.e., FDH) or transthyretin [11]. Therefore, prior to distinguishing TSHomas from RTHβ, we recommend heightened awareness of assay interferences, especially the potential involvement of FDH.

The coexistence of FDH and GD is uncommon. A few studies have briefly described cases of FDH combined with GD. Young reported a 50-year-old woman with concurrent FDH and GD in 1987 [12]. Jones reported that a 17-year-old female individual who presented with FDH and GD presented postpartum [13]. Khoo described a 42-year-old woman with both Hashimoto’s thyroiditis and GD in the setting of FDH [14]. Including the present case, all reported patients with FDH complicated by GD have been female. Regarding the treatment of GD in previous cases, one patient received RAI therapy and developed hypothyroidism, while two were prescribed antithyroid drugs. One patient quickly developed hypothyroidism, while the other achieved normal TSH levels within eight months.

In the present case, the treatment was more complex. Methimazole was initially administered but did not improve hyperthyroidism after one month. Because the patient was concerned that a longer course of methimazole therapy might delay her pregnancy planning, RAI therapy was then administered, and a thyroid function test after five months showed that the FT4 level was slightly elevated, the FT3 level was nearly within normal limits, and the TSH level was still significantly reduced. Furthermore, because the patient needed to prepare for pregnancy as soon as possible, a second RAI therapy was administered, and hyperthyroidism was eventually ameliorated. The patient developed hypothyroidism after receiving RAI therapy for GD. Because of the undiagnosed FDH, thyroid function failed to normalise despite frequent levothyroxine adjustments. In one instance, levothyroxine therapy was inappropriately discontinued at the local hospital. In fact, the levothyroxine dosage should only be adjusted based on TSH levels in patients with coexisting FDH and hypothyroidism [14]. Overall, from this case we learned that early recognition of FDH is helpful for the management of concomitant GD or hypothyroidism.

The patient in this case report was a woman of childbearing age with reproductive aspirations. It is generally believed that patients with FDH can become pregnant without any special intervention. For pregnancy complicated by FDH, excluding thyrotoxicosis is critical to avoid unnecessary treatment and its impact on the foetus [15]. A recent study found that the R218S variant of FDH might be related to high miscarriage rates [16], suggesting that pregnant patients with R218S mutation should be alert to adverse pregnancy outcomes.

In conclusion, FDH causes a similar pattern of thyroid hormone biochemistry to that of TSHomas and RTHβ and is an underdiagnosed cause of assay interference. Therefore, it is important to pay more attention to FDH when analysing the causes of IST. The coexistence of FDH and GD is rare and significantly complicates the disease management. Failure to identify FDH early can easily lead to misinterpretation of the thyroid function status and inappropriate treatment.

## Data Availability

All data generated or analysed during this study are included in this published article.

## Abbreviations

FDH: Familial dysalbuminemic hyperthyroxinemia
*ALB*: Albumin gene
T4: Thyroxine
TT4: Total thyroxine
RTH: Thyroid hormone resistance syndrome
TSH: Thyroid stimulating hormone
TSHomas: TSH-secreting pituitary adenomas
GD: Graves’ disease
RAI: Radioactive iodine
FT4: Free thyroxine
FT3: Free triiodothyronine
TT3: Total triiodothyronine
TRAb: Anti-TSH receptor antibody
mCi: Millicurie
SHBG: Sex hormone binding globulin
MRI: Magnetic resonance imaging
IST: Inappropriate secretion of thyroid stimulating hormone
RTHβ: Resistance to thyroid hormone β
